# Impact of Annual Health Checkups on Five-Year Weight Gain in Japan: Considering Behavioral Change Stages in the Transtheoretical Model

**DOI:** 10.7759/cureus.79655

**Published:** 2025-02-25

**Authors:** Yuri Akamatsu, Toshiyuki Ojima, Yoshitaka Nishikawa, Mayumi Toyama, Yoshimitsu Takahashi, Takeo Nakayama

**Affiliations:** 1 Department of Community Health and Preventive Medicine, Hamamatsu University School of Medicine, Hamamatsu, JPN; 2 Department of Health Informatics, Kyoto University School of Public Health, Kyoto, JPN; 3 Department of Implementation Science in Public Health, Kyoto University School of Public Health, Kyoto, JPN

**Keywords:** health check-up examination, obesity, stages of change, weight control, weight gain

## Abstract

Background

Obesity is a critical public health issue because this common disease leads to increased mortality. Therefore, controlling weight is essential. We aimed to evaluate whether undergoing health checkup examinations at least annually leads to better weight management.

Methodology

This longitudinal study utilized the health checkup examination data collected between April 2014 and March 2019 at the Seirei Health Care Division in Japan. Participants whose weight was measured in 2014 and 2019 were included. All analyses were performed according to sex and the three categories of health checkup examination frequency (twice, three to four times, and five to six times over five years). One-way analysis of variance (ANOVA) and trend analysis were conducted on the average weight differences between 2014 and 2019. Covariance analysis was performed using age, body mass index, and the categories of the behavioral change stages in the transtheoretical model in 2014 as covariates in the one-way ANOVA. Subgroup analyses were conducted for two age groups: ≥ 65 and < 65 years.

Results

A total of 84,078 males and 51,418 females were included. The mean age (standard deviation) in 2014 was 44.2 (13.1) and 46.7 (13.2), respectively. The more frequently weight was measured, the less weight was gained after five years in both sexes; the average weight gain was 1.32, 1.17, and 0.95 kg in males and 1.26, 0.96, and 0.78 kg in females in the twice, three to four times, and five to six times frequency groups, respectively. The *P*-values for the one-way ANOVA, trend analysis, and covariance analysis were all < 0.05. This trend was true for those aged <65 years, whereas in those aged ≥65 years, weight loss was noticeable.

Conclusions

An association between weight gain after five years and the frequency of health checkup examinations was revealed among individuals aged <65 years. Weight gain may be controlled with annual health checkup examinations.

## Introduction

Obesity is a major public health concern worldwide. According to the World Health Organization, one in eight adults aged ≥18 years were obese in 2022, and the prevalence of obesity more than doubled between 1990 and 2022 [1]. Moreover, obesity leads to increased mortality through complications such as cardiovascular disease, some types of cancers, hypertension, diabetes mellitus, and dyslipidemia [2,3]. Weight control is essential: weight loss for people with obesity and weight maintenance for people with healthy weights.

Effective ways of weight management include self-monitoring, which mainly consists of recording intake, physical activity, and weighing [4]. Through self-monitoring, people become more aware of their behavioral or activity patterns and thus can change their lifestyles [5]. Among the ways of self-monitoring, recording weight or self-weighing is easy to do by oneself, costs less, and is not accompanied by adverse psychological events [6-8]. Frequent self-weighing is favorable for weight management [4,7,9,10], but regular weight management is very difficult to maintain [6,11].

Japan has three main health checkup systems. First, employers are legally obliged to have their employees undergo regular health checkup examinations at least annually [12]. The regular health checkup examination includes 11 items, including an examination of height and weight and blood and urine tests [13]. Second, all people aged 40-74 years have the right to undergo *Specific Health Checkups (SHCs)* annually. The SHC system was initiated in 2008, and it aims to detect diseases related to metabolic syndrome in their earlier stages. In SHCs, examinations are performed as per regular health checkup examinations [14-16]. Third, in Japan, the Comprehensive Health Checkup System (Ningen Dock) was established and basically, those who undergo this belong to high socioeconomic status; they willingly undergo this to have diseases detected in their earlier stages or to maintain their health through prevention at their own expense or partially financially subsidized by the company [13,17-19]. In the Ningen Dock, people have more types of examinations than in regular health checkup examinations or SHCs. Therefore, in Japan, many people, especially those aged 40-74 years, undergo annual examinations for height and weight. In a sense, this health checkup examination is an opportunity for mandatory annual weighing, and this regular weighing is expected to have a similar effect on weight control as self-weighing. Hereafter, health checkup examinations refer to regular health checkup examinations, SHCs, and the Ningen Dock.

However, few studies have focused on the impact of the frequency of health checkup examinations [20,21]. The examinations are performed to maintain their health through prevention or detect diseases as soon as possible, and it is essential to research the impact. To our knowledge, there is no study focusing on the association between the frequency of examinations and weight control. Moreover, the previous studies have limitations where health awareness was not considered. This study aimed to evaluate whether the frequency of health checkup examinations is associated with weight control, considering health awareness.

## Materials and methods

Study design and setting

This longitudinal study utilized the health checkup examination and Comprehensive Health Checkup System (Ningen Dock) data collected in the Seirei Health Care Division (hereafter referred to as *Seirei*). Data were collected from April 2014 to March 2019 at five Seirei facilities in Shizuoka Prefecture: three facilities in Hamamatsu City and two in Shizuoka City. Hamamatsu City, located in the western part of Shizuoka Prefecture, has the largest population in Shizuoka Prefecture and is one of the government ordinance-designated cities. Shizuoka City is located in the central part of Shizuoka Prefecture and is its capital. All examinees are required to submit the questionnaire before they undergo the health checkup examination or Ningen Dock at Seirei.

Eligibility of participants

Participants whose weight was measured during health checkup examinations in 2014 and 2019 were included. Those whose age as of 2014 was not known were excluded.

Variables

We used the first data recorded in a year if the participants underwent a health checkup examination twice or more in the same year. The number of times the participants underwent health checkup examinations between 2014 and 2019 was categorized as twice, three to four times, and five to six times.

The health checkup examination questionnaire included the following question (Appendices A-C), based on the stages of change in the transtheoretical model [22]: “Do you want to improve your lifestyle habits of eating and exercising?” Each answer is equivalent to the stage of change: “Do not want to” corresponds to the pre-contemplation phase, “Do want to (within 6 months)” corresponds to the contemplation phase, “Want to improve in the near future (within a month)” corresponds to the preparation phase, “Already trying to improve (less than 6 months)” corresponds to the action phase, and “Already trying to improve (over 6 months)” corresponds to the maintenance phase. We divided these five phases into three categories, referring to previous studies [23,24]: the pre-contemplation, contemplation, and preparation phase, the action phase, and the maintenance phase. The outcome was the difference in body weight between 2014 and 2019.

Statistical analysis

We described the participants’ characteristics by sex and the frequency of health checkup examinations, the mean and standard deviation (SD) of age and body mass index (BMI), and the proportion of each category of the stages of change.

The average and SD of the difference in body weight between 2014 and 2019 were calculated according to sex and the three categories of the stages of change. One-way analysis of variance (ANOVA) was performed between the categories of the frequency of health checkup examinations according to sex. Trend analysis was also performed to verify whether weight gain monotonically decreased as the frequency of health checkup examinations increased. The interaction between the number of times participants underwent a health checkup examination between 2014 and 2019 and the age group with a cutoff of 65 years for population aging rate criteria (<65 years and ≥65 years) was calculated using two-way ANOVA. Analysis of covariance (ANCOVA) was performed by inserting age, BMI, and the categories of the stages of change in 2014 as covariates into the one-way ANOVA according to sex. Additionally, considering the effect of weight loss due to frailty [25-29], one-way ANOVA and ANCOVA were also conducted by age group (<65 years old and ≥65 years).

IBM SPSS Statistics, version 26 (IBM Corp., Armonk, NY) was used for all statistical analyses.

## Results

In total, 84,078 males and 51,418 females were included in this analysis, and the number of those whose age as of 2014 was not known was zero. Table 1 shows the characteristics of the participants in 2014 according to sex, age group, and the number of times they underwent health checkup examinations between 2014 and 2019. For males and females, the largest number of participants had health checkup examinations almost every year (five to six times in total) at 93.9% (78,991/84,078) for males and 89.8% (46,190/51,418) for females. This trend was similar by age group. The mean age (standard deviation) was 44.2 (13.1) in all males and 46.7 (13.2) in all females; it was approximately 44 years in males, regardless of the frequency of health checkup examinations, whereas in females, it was higher in the group who underwent health checkup examinations five to six times (46.9 years) than in the other groups (approximately 45 years). The average BMI was almost the same for each sex, regardless of the frequency of health checkup examinations: approximately 23 kg/m^2^ in males and 21.5 kg/m^2^ in females. Regarding the stages of change, the largest proportion of participants were in the contemplation and preparation phases among all individuals aged <65 years for both sexes, whereas the largest proportion were in the action and maintenance phases among individuals aged ≥65 years (Appendix D).

**Table 1:** Participants’ characteristics in 2014 by sex and frequency of health checkup examinations. There are no missing values for age. *NA expresses the number of missing values. BMI, body mass index; SD, standard deviation

Frequency of health checkup examinations between 2014 and 2019		n	Age (years)	BMI (kg/m^2^)		Stages of change					
						Pre-contemplation		Contemplation/preparation		Action/maintenance	
			Average (SD)	Average (SD)	NA*	*n* (%)		*n* (%)		*n* (%)	
All											
Males											
	Twice	696	44.2 (13.1)	23.4 (3.6)	1	138	(20.2)	370	(54.3)	174	(25.5)
	3 to 4 times	4391	44.7 (13.4)	23.4 (3.5)	4	851	(19.6)	2326	(53.7)	1154	(26.6)
	5 to 6 times	78991	44.1 (13.1)	23.2 (3.5)	63	16934	(21.8)	39372	(50.8)	21267	(26.9)
Females											
	Twice	637	45.0 (12.4)	21.7 (3.6)	1	71	(11.3)	408	(65.2)	147	(23.5)
	3 to 4 times	4591	44.6 (13.1)	21.5 (3.5)	2	647	(14.3)	2752	(60.8)	1127	(24.9)
	5 to 6 times	46190	46.9 (13.2)	21.6 (3.6)	16	6460	(14.2)	26412	(58.3)	12467	(27.5)
<65 years old											
Males											
	Twice	642	42.2 (11.4)	23.5 (3.6)	1	125	(19.5)	351	(54.7)	155	(24.1)
	3 to 4 times	4039	42.5 (11.6)	23.5 (3.6)	4	780	(19.3)	2211	(54.7)	989	(24.5)
	5 to 6 times	73289	42.2 (11.5)	23.3 (3.5)	61	15963	(21.8)	37735	(51.5)	18288	(25.0)
Females											
	Twice	596	43.3 (10.9)	21.7 (3.6)	1	64	(10.7)	392	(65.8)	129	(21.6)
	3 to 4 times	4259	42.7 (11.4)	21.4 (3.5)	1	585	(13.7)	2643	(62.1)	973	(22.8)
	5 to 6 times	41856	44.6 (11.4)	21.6 (3.6)	14	5754	(13.7)	24933	(59.6)	10405	(24.9)
≥65 years old											
Males											
	Twice	54	68.9 (4.6)	22.8 (2.5)	0	13	(24.1)	19	(35.2)	19	(35.2)
	3 to 4 times	352	69.5 (4.6)	22.7 (2.8)	0	71	(20.2)	115	(32.7)	165	(46.9)
	5 to 6 times	5702	69.3 (4.4)	23.2 (3.5)	2	971	(17.0)	1637	(28.7)	2979	(52.2)
Females											
	Twice	41	69.5 (5.2)	21.6 (2.9)	0	7	(17.1)	16	(39.0)	18	(43.9)
	3 to 4 times	332	69.2 (5.0)	22.0 (3.3)	1	62	(18.7)	109	(32.8)	154	(46.4)
	5 to 6 times	4334	69.6 (5.4)	21.8 (3.1)	2	706	(16.3)	1479	(34.1)	2062	(47.6)

Table 2 shows that among all ages for both sexes, the more frequently the participants had health checkup examinations, the smaller the amount of weight gain from 2014 to 2019 was. In males, the average weight gain was 1.32 kg in the *twice* frequency group and 0.95 kg in the *five to six times* frequency group, and in females, the average was 1.26 kg in the *twice* frequency group and 0.78 kg in the *five to six times* frequency group. One-way ANOVA and trend analysis resulted in *P* < 0.001 for males and females. Among people aged <65 years, the results were similar. However, among people ≥65 years of age, weight loss was observed in most categories, and no obvious trends were found. The subgroup effects of the number of times participants had health checkup examinations between 2014 and 2019 were significantly different from each other for the age subgroup (<65 and ≥65 years) (*P* for interaction < 0.001). 

**Table 2:** Association between the amount of weight gain and health checkup examination frequency. *Weight gain is calculated from the weight difference between 2014 and 2019. †*P*-value and *F*-value are the results of ANOVA. ‡*P*-value and JT statistic are the results of trend analysis. SD, standard deviation; ANOVA, analysis of variance; JT, Jonckheere-Terpstra

Frequency of health checkup examinations between 2014 and 2019	Males				Females			
	n	Weight gain* (kg)	*P*-value†	*P*-value‡	n	Weight gain* (kg)	*P*-value†	*P*-value‡
		Average (SD)	(*F*-value)†	(JT statistics)‡		Average (SD)	(*F*-value)†	(JT statistics)‡
All								
Twice	696	1.32 (4.61)	<0.001 (6.560)	<0.001 (-3.486)	637	1.26 (4.09)	<0.001 (8.414)	<0.001 (-3.631)
3 to 4 times	4391	1.17 (4.65)			4591	0.96 (4.65)		
5 to 6 times	78991	0.95 (4.33)			46190	0.78 (4.33)		
<65 years old								
Twice	642	1.48 (4.65)	<0.001 (7.585)	<0.001 (-3.951)	596	1.30 (4.11)	<0.001 (7.208)	<0.001 (-3.432)
3 to 4 times	4039	1.30 (4.73)			4259	1.09 (3.84)		
5 to 6 times	73289	1.06 (4.39)			41856	0.90 (3.60)		
≥65 years old								
Twice	54	-0.65 (3.48)	0.790 (0.235)	0.691 (0.397)	41	0.61 (3.58)	0.013 (4.335)	0.216 (1.238)
3 to 4 times	352	-0.34 (3.26)			332	-0.72 (3.22)		
5 to 6 times	5702	-0.40 (3.08)			4334	-0.36 (3.02)		

Table 3 shows that even after accounting for age, BMI, and stages of change categories using ANCOVA, an association remained between the frequency of health checkups and the amount of weight gain, as observed in the one-way ANOVA. Among participants younger than 65 years, the average weight gain was higher than that of the overall group, with a statistically significant association. In males, the average weight gain was 1.47 kg in the *twice* frequency group and 1.06 kg in the *five to six times* frequency group. In females, the average weight gain was 1.30 kg in the *twice* frequency group and 0.93 kg in the *five to six times* frequency group. Among people ≥ 65 years of age, similar to the ANOVA result, weight loss was observed in most categories, and no obvious trends were found.

**Table 3:** Association between weight gain and health checkup frequency, adjusted for covariates. Covariance analysis was performed, adjusting for age, body mass index (BMI), and the three categories of the stages of change, except in the groups of all ages and those younger than 65 years among males. In these groups, covariance analysis was adjusted for BMI and the stages of change categories, as there was an interaction between health checkup frequency and age. The *P*-value and *F*-value represent the results of the covariance analysis. *Weight gain is calculated from the weight difference between 2014 and 2019 for the age subgroups (<65 and ≥65 years). SD, standard deviation

Frequency of health checkup examinations between 2014 and 2019		Males			Females		
		n	Weight gain* (kg)	*P*-value (*F*-value)	n	Weight gain* (kg)	*P*-value (*F*-value)
			Average (SD)			Average (SD)	
All ages							
	Twice	682	1.31 (4.59)	<0.001 (8.96)	626	1.25 (4.09)	0.011 (4.47)
	3 to 4 times	4331	1.16 (4.66)		4526	0.97 (3.82)	
	5 to 6 times	77517	0.95 (4.33)		45331	0.78 (4.53)	
<65 years old							
	Twice	631	1.47 (4.64)	<0.001 (10.61)	585	1.30 (4.12)	0.013 (4.34)
	3 to 4 times	3980	1.29 (4.74)		4201	1.10 (3.83)	
	5 to 6 times	71930	1.06 (4.39)		41085	0.93 (3.58)	
≥65 years old							
	Twice	51	-0.66 (3.45)	0.095 (0.31)	41	0.62 (3.58)	0.011 (4.54)
	3 to 4 times	351	-0.33 (3.26)		325	-0.70 (3.22)	
	5 to 6 times	5587	-0.42 (3.07)		4246	-0.36 (3.01)	

## Discussion

In our study, we found that the more frequently the participants undertook health checkup examinations, the smaller the amount of weight gain among those <65 years of age, considering health awareness. To our knowledge, this is the first study to examine the association between weight-gain control and the frequency of health checkup examinations. Moreover, this study showed the result, considering health awareness, although the previous studies had the limitation of not considering it [20].

The finding in this study was consistent with that of previous studies examining the effect of regular self-weighing [4,7,9,10]. However, in the studies, the frequencies were daily or monthly. This study suggests that even annual weighing is effective in weight control, which is novel. The effect of regular self-weighing for weight control has been hypothesized to be that people become aware of their lifestyle behaviors and try to change them through regular self-weighing [5]. When this hypothesis is applied to our study, the following two mechanisms are expected. First, regular health checkup examinations are an opportunity to review their lifestyle behaviors or weight itself; individuals may review them when they see the results of their weight measurement or lab data at the time of health checkups or before the checkups so that their results are worse. Second, we must consider the effect of the opportunity to discuss health with medical staff and physicians, for example, at the examination or at the specific health guidance after SHC provided by specialists such as public health nurses [13-18]. Physician examination is essential for any type of health checkup examination. Specific guidance is not provided for all participants undergoing SHCs but rather for participants estimated to be at high risk of metabolic syndrome based on the SHC data [14,16,30,31]. Previous studies have revealed that discussing health with medical staff or motivational interviewing can lead to weight loss [32-34]. However, this is considered ineffective on an annual basis. This study suggests that annually discussing health with medical staff can have a long-term effect on weight control, although the effect may be small.

In this study, when age was divided into two groups (<65 and ≥65 years), the association between the frequency of health checkup examinations and weight gain was only revealed in the <65 years age group. This can be attributed to two main factors. First, there is an increase in the prevalence of frailty among people aged ≥65 years [25-27], and weight loss is one of the main symptoms of frailty [28,29]. The prevalence of frailty among Japanese individuals aged ≥65 years is reported to be 7.4%, with a prevalence of approximately 2% in the late 60s, which increases dramatically, reaching approximately 20% in the early 80s [25]. Second, there is an increasing incidence rate of diseases that lead to weight loss, such as cancer [35]. In this study, the number of people who underwent health checkup examinations only twice between 2014 and 2019 was very small. Considering that dieting is controversial in older people with obesity or overweight [33], there should be a focus on the possibility of weight control through frequent health checkup examinations among people < 65 years of age.

This study had several limitations. First, we only showed the association between the frequency of health checkup examinations and the amount of weight gained after five years, and caution should be taken when determining the direction of causality. Second, this study had selection bias because participants who had never undergone a health checkup examination or had the examination either in 2014 or 2019 were excluded. Furthermore, most participants aged ≥65 years were estimated to be retired, and these types of health checkup examinations are not mandatory. Among people aged ≥65 years, those who voluntarily underwent health checkup examinations both in 2014 and 2019 were included in this study, which may have led to selection and survival biases. Third, we did not consider lifestyles at baseline and time-dependent covariates, such as annual weight gain or loss, changes in lifestyles, including self-weighing, the occurrence of diseases affecting the change in weight (such as malignancy), and socioeconomic status, including occupation. Fourth, what type of health checkup examinations (the regular health checkup examinations, SHCs, or Ningen Dock) was not included in the data set of this study? It is possible that the results differed depending on the type of health checkup examinations, considering the characteristics of each examination. Finally, the proportion of those who received specific health guidance was not clear in this study, and the additional effect of the guidance on weight control could not be estimated.

## Conclusions

This study concludes that annual health checkup examinations can help control weight gain in individuals under 65. It also highlights that the health checkup examination system may be beneficial for weight management in the broader population that undergoes regular examinations.

The findings indicate that Japanese individuals under 65 who underwent more frequent assessments of their body weight during health checkup examinations managed to control their weight better over five years, even after accounting for health awareness. This conclusion is drawn from health checkup examinees data in Japan between fiscal years 2014 and 2019. Further research should be conducted to examine whether regular health checkup examinations can prevent obesity or its progression using different data.

## Figures and Tables

**Figure 1:**
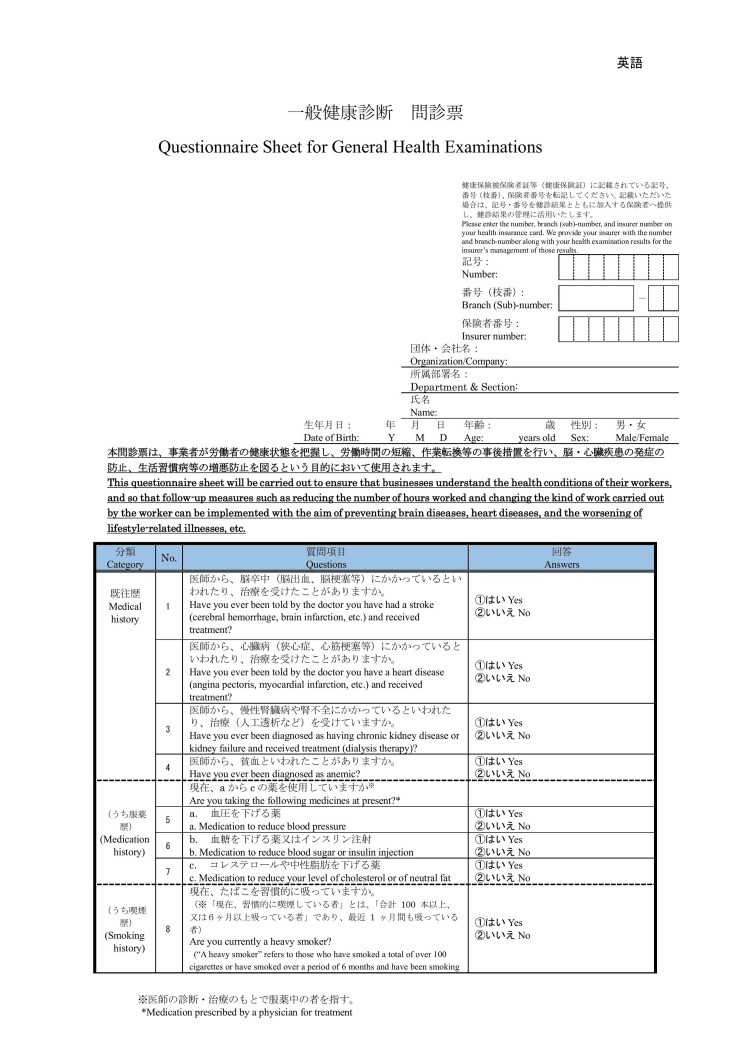
The questionnaire form commonly used in health checkup examinations in Japan (Page 1 of 3). This questionnaire form for health check-up examinnees was created and published online by Ministry of Health, Labour and Welfare, Japan. https://www.mhlw.go.jp/content/000793417.pdf

**Figure 2:**
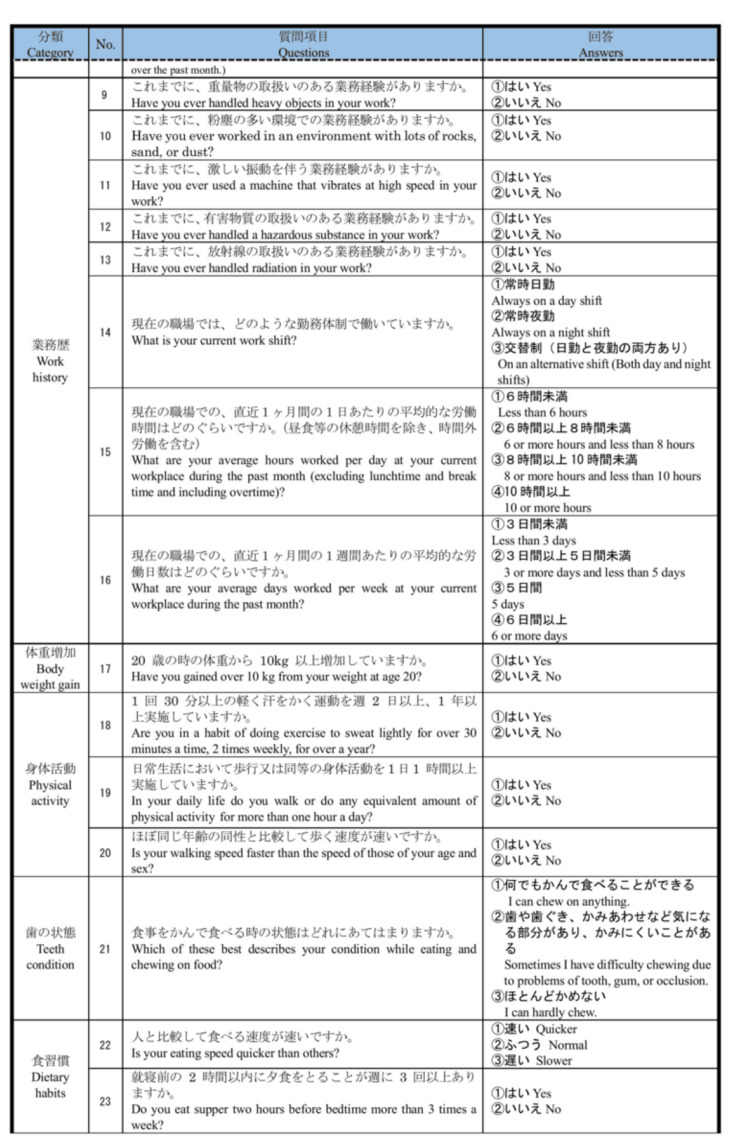
The questionnaire form commonly used in health checkup examinations in Japan (Page 2 of 3). This questionnaire form for health check-up examinnees was created and published online by Ministry of Health, Labour and Welfare, Japan. https://www.mhlw.go.jp/content/000793417.pdf

**Figure 3:**
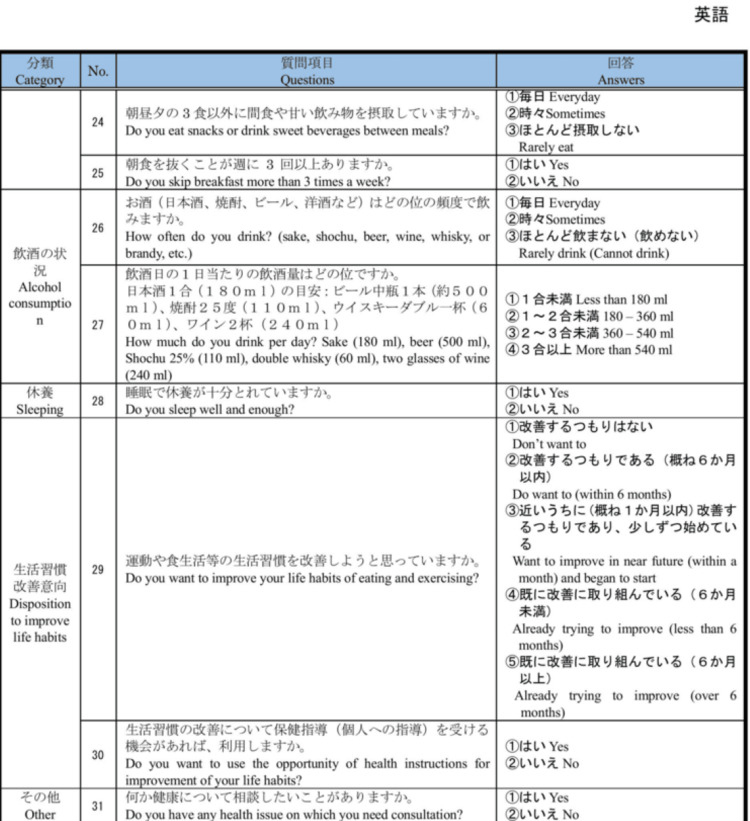
The questionnaire form commonly used in health checkup examinations in Japan (Page 3 of 3). This questionnaire form for health check-up examinnees was created and published online by Ministry of Health, Labour and Welfare, Japan. https://www.mhlw.go.jp/content/000793417.pdf

## Appendices

Appendix A 

Appendix B

Appendix C 

Appendix D

**Table 4:** Weight gain by sex, age groups, and the categories of behavioral change stages in the transtheoretical model. *Weight gain is calculated from the weight difference between 2014 and 2019. SD, standard deviation

Frequency of health check-up examinations between 2014 and 2019	Males				Females							
	n	Precontemplation	Contemplation/ preparation	Action/ maintenance	n	Precontemplation	Contemplation/ preparation	Action/ maintenance				
		Average of weight gain* (SD) (kg)				Average of weight gain* (SD) (kg)						
All												
twice	696	1.28 (4.41)	1.40 (4.84)	1.15 (4.41)	637	0.64 (4.14)	1.29 (4.05)	1.44 (4.17)				
3 to 4 times	4391	1.45 (4.60)	1.16 (4.84)	0.95 (4.33)	4591	0.86 (3.79)	1.04 (3.91)	0.86 (3.61)				
5 to 6 times	78991	1.27 (4.25)	0.94 (4.43)	0.73 (4.17)	46190	0.87 (3.53)	0.83 (3.61)	0.66 (3.47)				
< 65 years old												
twice	642	1.56 (4.35)	1.51 (4.80)	1.32 (4.47)	596	0.73 (4.25)	1.33 (4.09)	1.48 (4.15)				
3 to 4 times	4039	1.58 (4.70)	1.26 (4.87)	1.14 (4.47)	4259	1.10 (3.70)	1.10 (3.93)	1.09 (3.64)				
5 to 6 times	73289	1.37 (4.28)	1.00 (4.47)	0.91 (4.30)	41856	1.04 (3.49)	0.90 (3.63)	0.83 (3.56)				
≥ 65 years old												
twice	54	-1.34 (4.23)	-0.65 (3.18)	-0.22 (3.24)	41	-0.19 (3.16)	0.34 (2.67)	1.17 (4.43)				
3 to 4 times	352	-0.04 (2.90)	-0.73 (3.60)	-0.18 (3.15)	332	-1.38 (3.84)	-0.45 (3.11)	-0.60 (3.00)				
5 to 6 times	5702	-0.50 (3.18)	-0.49 (3.10)	-0.34 (3.02)	4334	-0.58 (3.53)	-0.43 (3.03)	-0.24 (2.79)				
